# 2354. Implementation of BPaL in the United States: Experience using a novel all-oral treatment regimen for rifampin-resistant or treatment-intolerant TB disease

**DOI:** 10.1093/ofid/ofac492.161

**Published:** 2022-12-15

**Authors:** Connie A Haley, David Ashkin, Charles A Peloquin

**Affiliations:** University of Florida, Nashville, Tennessee; University of Miami / Florida Health Department, Miami, Florida; University of Florida, Nashville, Tennessee

## Abstract

**Background:**

Tuberculosis (TB) remains a leading infectious cause of death and morbidity globally. Rifampin-resistance or intolerance requires prolonged treatment using less effective, more toxic regimens. Recent trials demonstrated that the all-oral six-month “BPaL” regimen, Bedaquiline, Pretomanid and Linezolid, is 90% effective. In these trials, linezolid-induced hematologic and neurologic toxicity was high using 1200mg daily, whereas lower exposure (dose or duration) reduced toxicity. Therapeutic drug monitoring (TDM) is used by U.S. TB experts to maintain a serum linezolid trough < 2ug/ml, which correlates with reduced toxicity. Since U.S. FDA approval in 2019, BPaL has been widely implemented for the treatment of rifampin-intolerant or resistant TB disease.

**Methods:**

We evaluated a cohort of patients with TB treated from October, 2019 through April, 2022, describing patient demographics, BPaL treatment dosing, and adverse events. TDM was performed for clinical purposes using liquid chromatography–mass spectrometry to measure serum levels at trough and 2 and 6h post-linezolid dose. Clinical providers adjusted linezolid dose and dosing interval targeting a trough < 2ug/ml and peak of 12–26ug/ml.

**Results:**

Among 64 BPaL patients, ages were 15–83 years, 22 (34.3%) were female, 6 (9.3%) U.S.-born, 4 (6.3%) HIV-infected. 50 (78.1%) had only pulmonary disease, 6 extrapulmonary, and 8 had both; 61 (91.0%) were culture-confirmed. Most (n=62) started linezolid 600mg daily. Linezolid was adjusted for 39 (66.1%) of the 59 patients with TDM; 18 had a trough >2ug/ml, 30 had dosing interval increased to thrice-weekly, and 17 had a dose increase. 52 (81.3%) patients completed BPaL and 12 remain on therapy. One 81-year-old female with diabetes, hypothyroidism, and B12 deficiency discontinued linezolid at 12 weeks for worsened neuropathy (linezolid trough=1.13ug/ml). She completed 26 weeks of bedaquiline/pretomanid and her symptoms returned to baseline.

**Conclusion:**

Use of BPaL with clinical and TDM monitoring has transformed treatment of rifampin-resistant or intolerant TB in the U.S. Patients previously sentenced to 18–24 months of treatment with 5–7 hard-to-tolerate medications and modest efficacy can now complete treatment in 6–9 months with little toxicity and exceptional cure rates.

**Disclosures:**

**All Authors**: No reported disclosures.

